# Validation and Assessment of Three Methods to Estimate 24-h Urinary Sodium Excretion from Spot Urine Samples in High-Risk Elder Patients of Stroke from the Rural Areas of Shaanxi Province

**DOI:** 10.3390/ijerph14101211

**Published:** 2017-10-11

**Authors:** Wenxia Ma, Xuejun Yin, Ruijuan Zhang, Furong Liu, Danrong Yang, Yameng Fan, Jie Rong, Maoyi Tian, Yan Yu

**Affiliations:** 1 Xi’an Jiaotong University Health Science Center, School of Public Health, No. 76 West Yanta Road, Xi’an 710061, Shaanxi, China; mawenxia1028@stu.xjtu.edu.cn (W.M.); zhangrj@mail.xjtu.edu.cn (R.Z.); 18792674133@163.com (F.L.); 18792423075@163.com (D.Y.); fym0401@stu.xjtu.edu.cn (Y.F.) rongjie5001@stu.xjtu.edu.cn (J.R.); 2 The George Institute for Global Health at Peking University Health Science Center, No. 6 Zhichun Road Haidian District, Beijing 100088, China; xyin@georgeinstitute.org.cn

**Keywords:** 24-h urine, spot urine, urinary sodium excretion

## Abstract

*Background*: 24-h urine collection is regarded as the “gold standard” for monitoring sodium intake at the population level, but ensuring high quality urine samples is difficult to achieve. The Kawasaki, International Study of Sodium, Potassium, and Blood Pressure (INTERSALT) and Tanaka methods have been used to estimate 24-h urinary sodium excretion from spot urine samples in some countries, but few studies have been performed to compare and validate these methods in the Chinese population. *Objective*: To compare and validate the Kawasaki, INTERSALT and Tanaka formulas in predicting 24-h urinary sodium excretion using spot urine samples in 365 high-risk elder patients of strokefrom the rural areas of Shaanxi province. *Methods*: Data were collected from a sub-sample of theSalt Substitute and Stroke Study. 365 high-risk elder patients of stroke from the rural areas of Shaanxi province participated and their spot and 24-h urine specimens were collected. The concentrations of sodium, potassium and creatinine in spot and 24-h urine samples wereanalysed. Estimated 24-h sodium excretion was predicted from spot urine concentration using the Kawasaki, INTERSALT, and Tanaka formulas. Pearson correlation coefficients and agreement by Bland-Altman method were computed for estimated and measured 24-h urinary sodium excretion. *Results*: The average 24-h urinary sodium excretion was 162.0 mmol/day, which representing a salt intake of 9.5 g/day. Three predictive equations had low correlation with the measured 24-h sodium excretion (r = 0.38, *p* < 0.01; ICC = 0.38, *p* < 0.01 for the Kawasaki; r = 0.35, *p* < 0.01; ICC = 0.31, *p* < 0.01 for the INTERSALT; r = 0.37, *p* < 0.01; ICC = 0.34, *p* < 0.01 for the Tanaka). Significant biases between estimated and measured 24-h sodium excretion were observed (all *p* < 0.01 for three methods). Among the three methods, the Kawasaki method was the least biased compared with the other two methods (mean bias: 31.90, 95% Cl: 23.84, 39.97). Overestimation occurred when the Kawasaki and Tanaka methods were used while the INTERSALT method underestimated 24-h sodium excretion. *Conclusion*: The Kawasaki, INTERSALT and Tanaka methods for estimation of 24-h urinary sodium excretion from spot urine specimens were inadequate for the assessment of sodium intake at the population level in high-risk elder patients of stroke from the rural areas of Shaanxi province, although the Kawasaki method was the least biased compared with the other two methods.

## 1. Background

Dietary sodium intake is associated with important cardiovascular disease risk factors such as hypertension [1]. It has been estimated that excess sodium intake accounts for 1.65 million deaths worldwide in 2010 [2]. In many countries, the average daily intake of salt is generally between 9 grams (g) and 12 g [3,4], whereas the recommended level for adults is less than 5 g of salt per day by the World Health Organization (WHO) [5]. Hence, WHO sets a target of a 30% relative reduction in mean population intake of salt by 2025 [6]. A continuous monitoring of the mean population sodium intake is essential for tracking the WHO salt reduction target. Because approximately 90% of ingested sodium is excreted in the urine, an accurate 24-h urine collection and measurement is crucial to determine the sodium intake. However, 24-h urine collection is burdensome for both participants and researchers, which leads to incomplete data and low response rate in surveys [7]. A previous study showed a random spot urine sample had minimal impact on the circadian patterns of the 24-h urinary sodium excretion [8]. Using spot urine has been explored as a promising method to estimate the 24-h urinary sodium excretion in many studies, including the Kawasaki method [9,10], the INTERSALT method [11], and the Tanaka method [8]. However, few studies have been conducted in the Chinese population. This study aims to compare and validate the Kawasaki, INTERSALT, and Tanaka formulas, to predict 24-h urinary sodium excretion using random spot urine samples in a rural Chinese population in Shaanxi province.

## 2. Methods

Data were collected from a sub-sample of the ongoing Salt Substitute and Stroke Study (SSaSS) [12]. SSaSS is a large-scale cluster randomized controlled trial conducted in five provinces in Northern China. The trial (Registration number at Clinical Trials.gov identifier: NCT02092090) has been approved by the Ethics Committee of University of Sydney, Peking University Health Science Center and Xi’an Jiaotong University Health Science Center. Written informed consent has been obtained from all study participants.

### 2.1. Sites

The design of the SSaSS had been described elsewhere [12]. Briefly, Shaanxi was one of the five provinces participating in this trial. 120 villages from Chenggu and Qishan County (60 villages in each county) were recruited in Shaanxi province. 120 villages were randomized into intervention or control villages in a 1:1 ratio with stratification by county. Therefore, in each county, 30 intervention villages and 30 control villages participated. At baseline and every year after randomization, 10% of the villages in each group were randomly selected to participate in the process. Overall, in each county, there were three randomly-selected intervention villages and three randomly-selected control villages that participated in the process indicator survey in each county.

### 2.2. Subjects

In each village, about 35 participants with elevated risk of stroke were enrolled and were followed-up every six months for five years. The definition of the elevated risk of stroke was on the basis of a history of prior stroke and/or age 60 years or above with uncontrolled high blood pressure (BP) (systolic BP ≥ 140 mm Hg at visit if on BP lowering medication or systolic BP ≥ 160 mm Hg if not on BP lowering medication). At the baseline and one-year post-randomization process indicator survey, which occurred in Feb 2015 and Feb 2016 respectively, 20 participants were randomly selected from each participating village to participate. All participants were invited to complete a structured questionnaire including demographic information, physical measurements and BP measurement. Face-to-face interviews were conducted by trained medical students from the School of Public Health of Xi’an Jiaotong University Health Science Center. Right arm BP was repeatedly measured by standardized electronic sphygmomanometer (HEM-7301-IT, Omron Corporation, Kyoto, Japan) following standardized methods, in a sitting position after five min of rest and the time interval between successive pairs of BP measurements was two min. Participants with the following conditions were not required to provide urine samples. (1) had difficulty in collecting urine sample (i.e., urinary incontinence or body difficulties without other people to assist them in the urine collection); (2) acute and chronic urinary infection; (3) pregnant, breastfeeding, menstruating (individuals are only eligible to be included two days after the menstruation ends); (4) vomit or diarrhoea.

### 2.3. Urine Collection

Eligible participants were suggested not to change their dietary and lifestyle habits deliberately and were provided with clean, unused containers to collect urine samples including a 50 mL cup for spot urine and 6 1-litre plastic buckets for 24-h urine. Participants were instructed verbally, and were given written instructions on how to collect the spot and 24-h urine samples. For elder participants, we given instructions to other members of the family as well and they were asked to provide support to the participants for collecting 24-h urine properly. Before the onset of 24-h urine collections, participants were asked to provide a clean midstream urine as spot urine specimens. Participants were then instructed to void all urine in the subsequent 24 h into the provided buckets without omission. Participants were asked to return the 24-h urine on the next day, then the samples mixed in a 5 litre plastic container with a glass stick from which two 2 mL aliquots were taken and stored in the plastic Eppendorf tubes. Upon completion of the collection, the interviewers recorded the start and finishing time of the collection, total urine volume, and the missing urine volume, based on self-reported. For quality control purpose, we randomly selected three participants in each village to collect additional two aliquots with dummy numbers as the blinded samples. 24-h urine specimens with the following conditions were excluded: (1) missed the morning fasting urine; (2) missed more than 10% of the total volume; (3) contaminated with faeces or blood; (4) vomiting or diarrhoea during the collection. All the aliquots were frozen at −20 °C immediately after collection and shipped to the centre laboratory in Beijing within seven days.

### 2.4. Specimen Analysis

All samples were processed in the central laboratory hosted in the Civil Aviation General Hospital in Beijing. Sodium and potassium concentrations were determined by the direct ion-selective electrode method, while creatinine concentration was analysed using the enzymatic method. Analysis of these electrolytes were performed automatically with Hitachi 7600 auto-biochemistry clinical analyser (Hitachi, Tokyo, Japan). Quality-control measures included the quality control samples and 72 participants duplicate specimens of the same sample were marked with dummy identification numbers to blind the laboratory to the process.

Finally, we took measures to assess the completeness of 24-h urine collections, including assessment of the duration of the collection, the total urine volume and 24-h creatinine excretion. 24-h urine samples were excluded from analysis if the time of the collection fell outside the range of 22–26 h, if the total 24-h urine volume was less than 500 mL or greater than 6000 mL, and if 24-h creatinine excretion was less than 3 mmol or greater than 25 mmol in women or less than 6 mmol or greater than 30 mmol in men.

### 2.5. The Estimated 24-h Sodium Excretion from Spot Urine Samples

The measured 24-h urinary sodium excretion (mmol/day) = concentration of 24-h urinary sodium excretion (mmol/L) × 24-h urine volume (L/day).

The estimated values of 24-h urinary sodium excretion was calculated for each of the Kawasaki, INTERSALT and Tanaka methods that were listed in Table 1.

### 2.6. Statistical Analysis

All data were coded using Epidata version 3.1 (the Epidata association, Odense, Denmark). Statistical analysis was performed using SPSS 18.0 for Windows (SPSS & IBM, Inc., Chicago, IL, USA). The correlation of estimated against measured 24-h urinary sodium excretion was assessed by the Pearson correlation coefficient (r) and intraclass correlation coefficient (ICC) and visualized using scatter plots. The difference between measured and estimated 24-h urinary sodium excretion was tested by paired *t*-test. The Bland-Altman (BA) method was used to further explore the agreement between measured and estimated 24-h urinary sodium excretion. BA plots were generated by MedCalc version 17.6 (MedCalc Software bvba, Mariakerke, Belgium). Null hypotheses of no difference were rejected if the respective *p* < 0.01. All P values were 2-tailed.

## 3. Results

We took quality-control measures and the percentage of technical error was calculated and final values were 1.67% for sodium, 1.68% for potassium and 2.36% for creatinine at baseline survey, and 3.14% for sodium, 0.80% for potassium and 1.23% for creatinine at one-year process indicator survey.

### 3.1. Subject Characteristics

A total of 365 participants from the rural areas of Shaanxi province were included in the final analysis. 33 were excluded for missing either spot urine or 24-h urine. Mean 24-h urine volume was 1419.3 mL and mean collection time was 23.7 h. The average 24-h urinary sodium excretion was 162.0 mmol/day, which corresponded to a salt intake of 9.5 g/day (1 mg Na^+^ ≈ 2.54 mg NaCl) [13]. The characteristics of subjects were presented in Table 2.

### 3.2. Validity of Three Methods of Estimated versus Measured 24-h Urinary Sodium Excretion in High-Risk Elder Patients of Stroke from the Rural Areas of Shaanxi Province

#### 3.2.1. Correlation between Estimated and Measured Sodium Excretion

**Figure 1:**
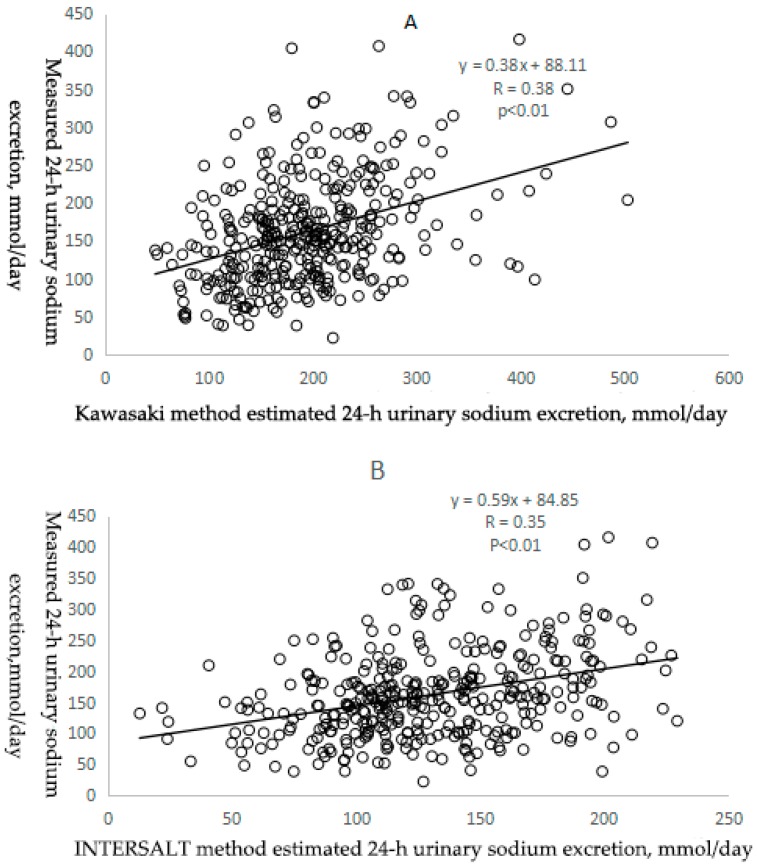

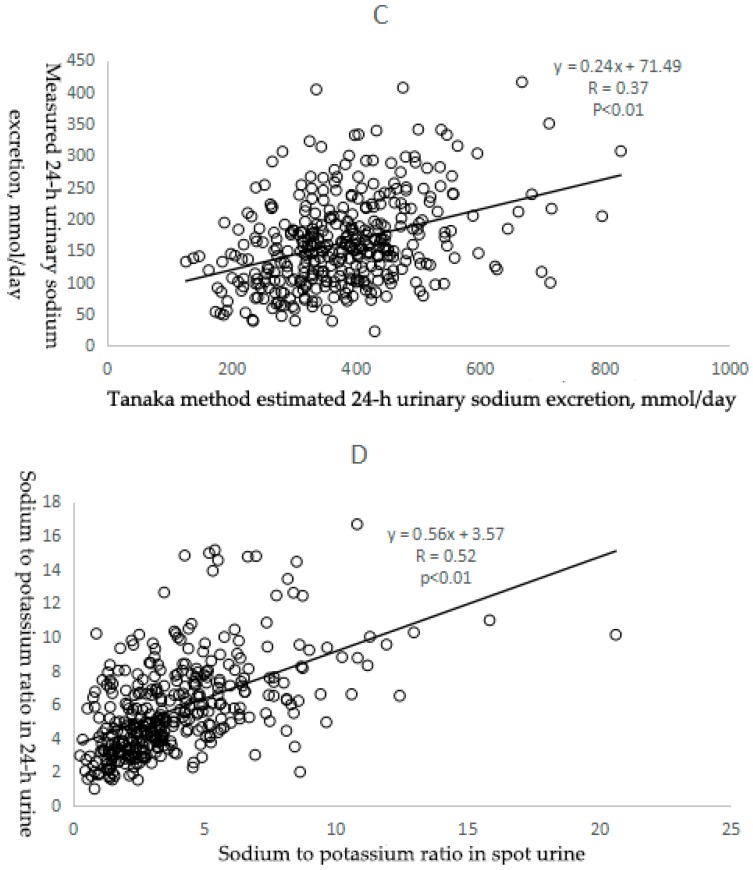
Scatter plots of measured 24-h urinary sodium excretion (mmol/day) versus the Kawasaki (**A**); INTERSAL (**B**); and Tanaka (**C**); methods estimated 24-h urinary sodium excretion (mmol/day) and scatter plot of Na/K ratio in spot urine versus Na/K ratio in 24-h urine. (**D**) The hollow circles were scatter points of measured and estimated values. The solid black line was the regression line of the scatters.

Pearson correlation coefficients between measured and estimated 24-h urinary sodium excretion were low, 0.38 for the Kawasaki method, 0.35 for the INTERSALT method and 0.37 for the Tanaka method (all *p* < 0.01) (Table 3 and Figure 1). In addition, ICCs showed a similar pattern as the person correlation coefficients, 0.38 (95% CI: 0.29, 0.47) for the Kawasaki method, 0.31 (95% CI: 0.21, 0.40) for the INTERSALT method and 0.34 (95% CI: 0.25, 0.43) for the Tanaka method (all *p* < 0.01) (Table 3).

As for the sodium to potassium (Na/K) ratio, sodium and potassium concentrations of spot urine versus 24-h urine values, the correlation coefficient (r = 0.52 and *p* < 0.01) (Table 4 and Figure 1D) and ICC (ICC = 0.52, 95% Cl: 0.44, 0.59 and *p* < 0.01) (Table 4) were highest compared with these three prediction equations.

#### 3.2.2. Bias between Estimated and Measured Sodium Excretion

Kawasaki method had the smallest bias among the three methods, with mean bias of 31.90 (95% CI: 23.84, 39.97) whereas the bias for the INTERSALT method and the Tanaka method were −32.04 (95% CI: −39.04, −25.04) and 216.72 (95% CI: 205.81, 227.65), respectively (all *p* < 0.01). Using Kawasaki method and Tanaka method, mean estimated 24-h urinary sodium excretion was higher compared to measured values (*t* = 7.78, *p* < 0.01; *t* = 39.03, *p* < 0.01). On the contrary, using INTERSALT method, mean estimated 24-h urinary sodium excretion was lower (*t* = −9.00, *p* < 0.01) (Table 3).

**Figure 2:**
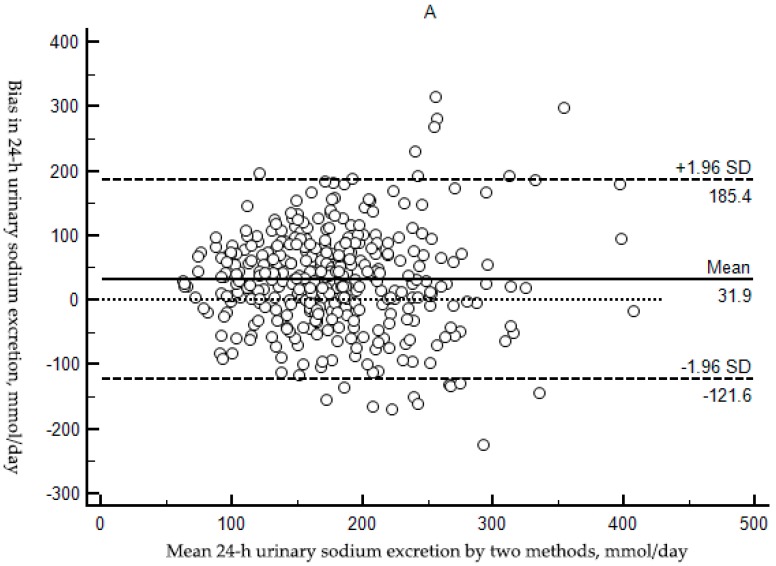

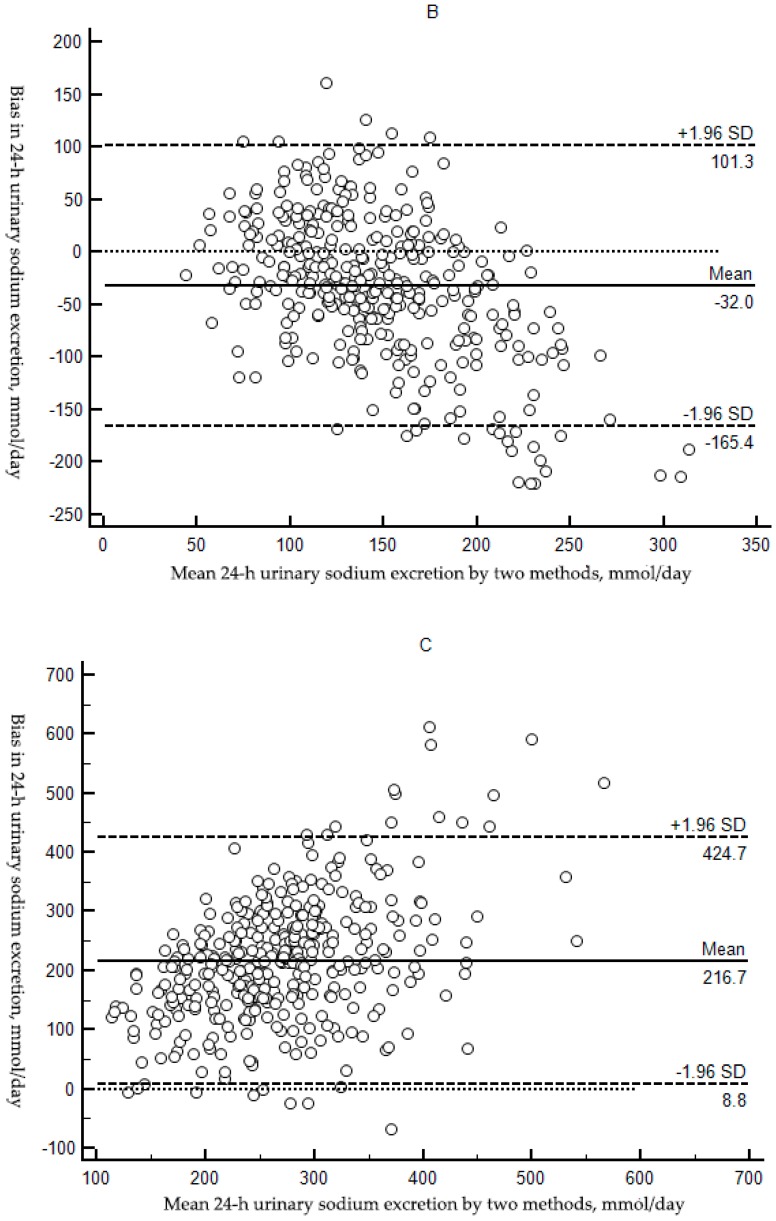

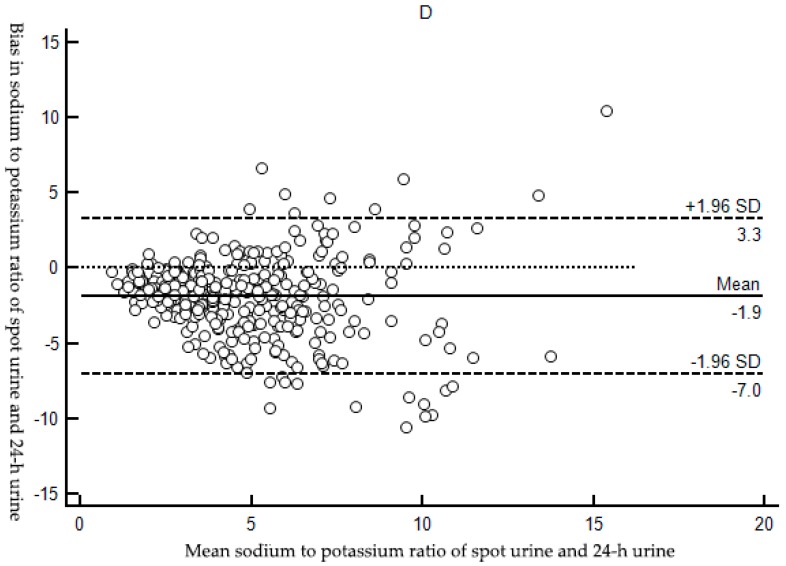
Bland-Altman plots of measured 24-h urinary sodium excretion (mmol/day) versus the Kawasaki (**A**); INTERSALT (**B**); and Tanaka (**C**); methods estimated 24-h urinary sodium excretion (mmol/day) and Bland-Altman plot of Na/K ratio in spot urine versus Na/K ratio in 24-h urine; (**D**) The horizontal axis represented mean ((estimated values plus measured value)/2), and the vertical axis represented difference (estimated values minus measured value). The solid blue line represented the mean of difference, the dashed black lines represented the 95% LoA of mean, and dashed red line represented that the difference was zero.

BA plots, visual representation of the agreement between two different techniques, were used to assess bias. There was significant tendency of over-and under-estimation depending on the bias. With the INTERSALT method, the mean estimated 24-h urinary sodium excretion levels were consistently underestimated relative to the measured true values (Figure 2B) and overestimation occurred when the Kawasaki and Tanaka method was used (Figure 2A,C) though the Kawasaki method performed a relative accuracy among the three methods: the least gap between the estimated and measured 24-h urinary sodium excretion among the three methods.

In the context of Na/K ratio, spot urine Na/K ratio were mostly lower than 24-h urine Na/K ratio and the mean bias was −1.85 (95% CI: −2.12,−1.58) (*p* < 0.01) (Table 4 and Figure 2D).

## 4. Discussion

Our study was to compare and validate the three predictive equations on the estimation of 24-h urinary sodium excretion using spot urine samples. We found that three methods (the Kawasaki, INTERSALT and Tanaka methods) were not valid to estimate 24-h sodium intake in high-risk elder patients of stroke from the rural areas of Shaanxi province, and Kawasaki method was found with the least bias compared with the other two methods.

It was worth noting that the incompleteness of 24-h urine collection may result in incorrect conclusion after applying these formulas. In our study, average urine volume was 1419.3 mL/day (SD: 547.3 mL/day), which similar to the value ((1442 ± 577) mL/day) reported in a related study carried out in Yantai, China in 2014 [14]. Similarly, Han, W. et al. [15] found mean intake of sodium was 9.0 g/day in Beijing in hypertension patients while Xu, J. et al. [14] computed an average salt intake of 11.8 g/day in YanTai in a general population. In consideration of the similar levels of urine volume and salt intake as well as the measures of assessing the completeness of 24-US collection, including assessment of 24-h creatinine excretion, 24-h urine volume, self-report or a combination of these factors, our study, therefore, seemed to show an accurate collection of a complete 24-h urine collection.

The Kawasaki and Tanaka methods were developed using a similar hypothesis: 24-h urinary sodium excretion estimated from spot urine was established based on a strong correlation of measured and predicted 24-h urinary creatinine excretion from weight, height and age as well as a good correlation of Na/Cr ratio between spot urine and 24-h urine [16]. In our study, we observed the correlation between predicted and measured creatinine excretion (r = 0.50, *p* < 0.01 for the Kawasaki method and r = 0.44, *p* < 0.01 for the Tanaka method, respectively) (Supplementary Materials Figure S1) and the correlation of Na/Cr ratio between spot urine and 24-h urine samples (r = 0.40, *p* < 0.01) (Supplementary Materials Figure S2), and concluded that spot urine samples were acceptable for estimating 24-h urinary sodium excretion. It was emphasized that the negligence of validation of creatinine excretion and Na/Cr ratio between spot urine and 24-h urine before applying these methods was likely to lead to improper use in the calculation of sodium excretion [17,18,19].

In our study, all the three methods were adjusted by the concentration of creatinine due to its consistent excretion in urine [20,21,22]. It has been accepted that agreement tests between methods need to be performed for evaluation if spot urine estimation method can be a substitute of 24-h urine collection method. The correlation between estimated and measured sodium excretion showed the three equations had fairly low correlation (r = 0.35–0.38, all *p* < 0.01; ICC = 0.31–0.38, all *p* < 0.01) to the measured values in our study. These results were similar with a sub-study of PURE study that reported a correlation coefficient of 0.19 for the Kawasaki and the INTERSALT methods and 0.29 for the Tanaka method and meanwhile, ICCs were moderated from 0.21 to 0.29 [13]. Accordingly, a study used the Tanaka method to adopt an estimation from random spot urine and reported a coefficient of 0.54 in Japanese population and 0.32 in a validation sample [8]. We had to admit that low correlation indicated that the three 24-h urinary sodium excretion estimation equations with spot urine samples may not be fit in our study population. However, correlation measured the strength of an association, just a part of evaluation of agreement between methods, so it was not enough. In this regard, the BA method was commonly explored to determine agreement between methods [23].

Our following was to explore the bias between estimated and measured sodium excretion and significant biases were identified for the three methods (all *p* < 0.01). We also found by BA plots that of the three predictive equations evaluated, estimation of sodium excretion from spot urine using the Kawasaki formula showed the least bias and most agreement with measured 24-h urinary sodium excretion (mean bias: 31.90 mmol/day, 95% Cl: (23.84,39.97) mmol/day). However, the Kawasaki method overestimated 24-h urinary sodium excretion while overestimation tended to occur for the Tanaka method and underestimation for the INTERSALT method, which was another important limitation why these methods cannot be worked as a substitute of 24-h urine collection method. It was worth noting that the results in our present study seemed to show consistent under- or over-estimation throughout low to high sodium ranges whereas the PURE study illustrated that overestimation of 24-h urinary sodium excretion might be in lower sodium level while underestimation in higher level for the three methods [24]. It was possible that the inconsistencies could be explained by the different characteristics of subjects investigated and different method we used. For instance, the participants recruited in PURE study were from diverse populations in low, middle and high-income countries, representing a relatively broad distribution of age and different levels of economic. On the contrary, our subjects were adults aged 47–91 years from the rural areas of a single province. Obviously, the participants in former study were younger than our subjects (57 years vs 68 years) and had lower BP (131.1/81.6 mm Hg) than ours (169.6/95.5 mm Hg), expected to have entirely normal renal function. There were more hypertensive patients in our study, and patients may have a different diurnal pattern of sodium excretion than normotensive participants [25], which perhaps affected the basis and effectiveness of the predictive methods.

The aforementioned results confirmed that the Kawasaki, INTERSALT and Tanaka methods based on spot urine specimens were inadequate for assessment of sodium intake at the population level in high-risk elder patients of stroke from the rural areas of Shaanxi province. In addition, our data also confirmed that these methods could not be applied for individual to estimate their 24-h sodium excretion owing to the bias in individual estimated 24-h sodium excretion across low to high sodium levels. With a consistency, our results were similar to the previous study [13]. However, a study in China, on the contrary, suggested the Kawasaki method provided reasonably accurate estimation [15]. In addition, some studies suggested that the sodium level in afternoon or evening spot urine specimens could better represent 24-h urinary sodium excretion [26,27] and it seemed that there was an optimum urine collection time for these predictive methods [19]. In our study, we took spot urine to estimate 24-h urinary sodium excretion. Future studies in Chinese population should include additional spot urine specimens.

As for the low accuracy of these estimation methods in high-risk elder patients of stroke from the rural areas of Shaanxi province, it was possible to had a relationship with ethnic difference. In general, the Kawasaki [9] and Tanaka equations [8] were developed from Japanese population, and the INTERSALT equation [11] was developed from North American and European population. In a word, all the three were not developed from Chinese population, so they were biased when equations applied to the population. Furthermore, it was emphasized that both the Tanaka and INTERSALT methods were developed and validated in a population of the young adults [8,11] and the Kawasaki method in populations of broader age spectrum [9,28] while the participants recruited in our study were adults aged 47–91 years from a single province. Moreover, it should be noted that the Kawasaki equation was initially developed for use with a second morning urine specimens [9]. It was plausible that these predictive methods may be suitable for estimation in different population, hence the validation studies in different populations would be required.

Another finding from our study was that we checked the method for estimating population 24-h urine Na/K ratio from Na/K ratio in spot urine suggested in the recent publication [29] and we observed high correlation (r = 0.52 and ICC = 0.52, respectively) and small bias (−1.85, 95% Cl: −2.12, −1.58) of Na/K ratio between 24-h urine and spot urine. With a consistency, our results saw some agreements with the study [29], which was based on INTERSALT study and included Chinese population. Besides, spot urine was randomly collected in the daytime in our study. Our date showed a lower daytime Na/K ratio in spot urine than Na/K ratio throughout the 24-h, which was consistent with the results [29] and lower Na/K ratio was also observed in the morning and afternoon than in the evening and overnight [30]. Previous studies suggested that food intake was not a significant contributor to the circadian rhythm in sodium and potassium excretion [31,32] while hormonal factors were mainly related to the fluctuation. The excretion of renal sodium and potassium was under the control of hormones, such as aldosterone [33,34]. High aldosterone secretion could induce a decrease in sodium excretion and an increase in potassium excretion [33] and aldosterone was inactive during sleeping time and active after arising in humans [27,35]. Therefore, the period of active sodium reabsorption would be reflected in the urine void after arising until daytime, causing relatively less sodium excretion and lower Na/K ratios. Hence, we speculated that we got less biased and much reliable data in Chinese population due to similar study population and similar results. This might explain our speculation about the low accuracy of the three predictive methods in our study population for no ethnic basis of Chinese population. Day-to-day variability and large circadian in the concentrations of sodium and potassium in spot urine, urine volume and voiding frequency possibly reduced the ability to estimate 24-h sodium and potassium excretion alone from spot urine specimens without use of demographic and additional urine variables [29]. The Kawasaki, INTERSALT and Tanaka methods used multiple variables, such as urinary creatinine, BMI, age and gender, and our date showed that these were not acceptable for assessment of mean sodium intake in our study population. There were not any variables in the estimation procedure other than Na/K ratio itself, as Na/K ratio was not dependent on urine volume and might also correct to some extent for body size [29], which made the estimation of Na/K ratio in 24-h urine from spot urine more straightforward than the predictive equations with use of demographic and additional urine variables. This method, however, has not been validated in diverse demographic groups. Further investigation was needed to address this issue.

Of course, the present study had limitations. Firstly, a single measured concentration level of 24-h urine sodium was not sufficient to assess the diurnal variability of sodium excretion within person between the initial and follow-up collection. Precision might be improved by getting more than one specimen per person and multiple measurements [36]. However, 24-h urine specimen was not often feasible due to high labour burden and difficulty in complete collection, which hence affected response rate and practicality of the test, especially in large epidemiological studies [37]. Secondly, creatinine concentration, a significant reference index for the Kawasaki, INTERSALT and Tanaka methods, was still an issue. Personal creatinine excretion varied daily due to the intake of dietary protein and physical activity though it often be considered to be relatively stable [38]. Taking these sensitive factors into account, we emphasized in our survey that participants should not change their dietary and lifestyle habits deliberately, indeed, it was difficult to control these participants within urine collection time. Thirdly, we just used small samples to assess the use of spot urine for estimating 24-h urinary sodium excretion, and precision could be greatly improved by using larger samples. Therefore, large-scale, population-based epidemiological researches were needed.

## 5. Conclusions

Estimation of 24-h urinary sodium excretion from spot urine by the Kawasaki, INTERSALT and Tanaka methods were inadequate for assessment of sodium intake at the population level in high-risk elder patients of stroke from the rural areas of Shaanxi province, although the Kawasaki method was the least biased compared with the other two methods. A more accurate method, based on our own population sodium excretion data, should be developed in future studies.

## Figures and Tables

**Table 1:** Three methods to estimate 24-h urinary sodium excretion from spot urine samples.

Method	Urine Sample	Formula to Estimate 24-h Urinary Sodium Excretion (mmol/day)
Kawasaki [9,10]	Second morning urine *	16.3 × (Naspot (mmol/L)/Crspot (mg/dL) × 1/10 × PrUCr24h (mg/day)) 0.5
		PrUCr24h = 15.12 × Weight (kg)+7.39 × Height (cm) − 12.63 × Age (year) − 79.90 (Male)
		PrUCr24h = 8.58 × Weight (kg) + 5.09 × Height (cm) − 4.72 × Age (year) − 74.50 (Female)
INTERSALT [11]	Casual spot urine	(25.46 + 0.46 × Naspot (mmol/L)) − 2.75 × Crspot (mmol/L) − 0.13 × Kspot (mmol/L) + 4.10 × BMI (kg/m^2^) + 0.26 × Age (year) (Male)
		(5.07 + 0.34 × Naspot (mmol/L)) − 2.16 × Crspot (mmol/L) − 0.09 × Kspot (mmol/L) + 2.39 × BMI (kg/m^2^) + 2.35 × Age (year) − 0.03 × Age 2 (year)) (Female)
Tanaka [8]	Casual spot urine	21.98 × (Naspot (mmol/L)/Crspot (mg/dL) × PrUCr24h (mg/day)) 0.392
		PrUCr24h = 14.89 × Weight (kg) + 16.14 × Height (cm) − 2.04 × Age (year) − 2244.45

Predicted 24-h urinary creatinine, PrUCr24h; Spot urinary sodium, Naspot; Spot urinary potassium, Kspot; Spot urinary creatinine, Crspot. * We replace the second morning urine with the casual spot urine.

**Table 2:** Demographic and clinical characteristics of the study population (*n* =365).

Selected Characteristics	Mean (SD) ^1^/*n* (%)
Age (years)	67.5 ± 6.8
Female	210 (57.5)
Weight (kg)	61.3 ± 10.5
Height (cm)	158.4 ± 7.8
Body mass index (kg/m^2^)	24.4 ± 3.5
Systolic blood pressure (mm Hg)	169.6 ± 24.0
Diastolic blood pressure (mm Hg)	95.5 ± 15.5
Hypertension ^2^	354 (97.0)
Spot Urine	
Sodium concentration (mmol/L)	130.4 ± 55.0
Potassium concentration (mmol/L)	44.9 ± 27.1
Creatinine concentration (mg/L)	1171.9 ± 677.4
24-h urine	
Sodium concentration (mmol/L)	122.4 ± 47.9
Potassium concentration (mmol/L)	24.7 ± 12.4
Creatinine concentration (mg/L)	730.5 ± 312.5
24-h urine volume (mL)	1419.3 ± 547.3
The duration of the 24-h urine collection (hour)	23.7 ± 0.5

^1^ SD: standard deviation; ^2^ Hypertension was defined by an average systolic BP ≥ 140 mm Hg or an average diastolic BP ≥ 90 mm Hg at physical examination or they were receiving anti-hypertensive drugs or they had a previous diagnosis of hypertension for at least two weeks.

**Table 3:** Validity of three methods of estimated versus measured 24-h urinary sodium excretion in high-risk elder patients of stroke from the rural areas of Shaanxi province.

Variables	Measured 24-h Urinary Sodium Excretion	Estimated 24-h Urinary Sodium Excretion		
		Kawasaki Method	INTERSALT Method	Tanaka Method
Sodium excretion (mmol/day)	162.02 ± 70.35	193.92 ± 70.66	129.97 ± 41.49	378.74 ± 109.90
Range of sodium excretion (mmol/day)	22.08–415.80	47.96–502.91	12.69–229.39	126.45–825.49
ICC (95% Cl) ^1^	Reference	0.38 (0.29, 0.47)	0.31 (0.21, 0.40)	0.34 (0.25, 0.43)
Correlation coefficient ^2^	Reference	0.38	0.35	0.37
Bias (mg/day, 95% Cl) ^3^	Reference	31.90 (23.84,39.97)	−32.04 (−39.04,−25.04)	216.72 (205.81,227.65)

^1^ We used the value of single measures and all *p* < 0.01; ICC: intraclass correlation coefficient; 95% Cl: 95% confidence interval; ^2^ all *p* < 0.01; ^3^ bias: estimated values minus measured values and all *p* < 0.01.

**Table 4:** Validity of Na/K ratio in 24-h urine versus Na/K ratio in spot urine in high-risk elder patients of stroke from the rural areas of Shaanxi province.

Variables	Na/K Ratio in 24-h Urine	Na/K Ratio in Spot Urine
Mean ± SD ^1^	5.73 ± 2.79	3.88 ± 2.62
ICC (95%Cl) ^2^	Reference	0.52 (0.44,0.59)
Correlation coefficient ^3^	Reference	0.52
Bias( 95%Cl) ^4^	Reference	−1.85 (−2.12,−1.58)

^1^ SD: standard deviation; ^2^ We used the value of single measures and *p* < 0.01; ICC: intraclass correlation coefficient; 95%Cl: 95% confidence interval; ^3^ *p* < 0.01; ^4^ bias: value of Na/K ratio in spot urine minus Na/K ratio in 24-h urine and *p* < 0.01.

## Supplementary Materials

The following are available online at www.mdpi.com/1660-4601/14/10/1211/s1, Figure S1: Scatter plots of measured 24-h creatinine excretion (mg/day) vs Kawasaki (A) and Tanaka (B) methods estimated 24-h creatinine excretion (mg/day). The hollow circles were scatter points of measured and estimated values. The solid black line was the regression line of the scatters, Figure S2: Scatter plots of the ratio of sodium to creatinine (mmol/mg) in spot urine vs the ratio of sodium to creatinine in 24-h urine (mmol/mg). The hollow circles were scatter points of the ratio values. The solid black line was the regression line of the scatters.
