# Supplementary material for: Validation and Assessment of Three Methods to Estimate 24-h Urinary Sodium Excretion from Spot Urine Samples in High-Risk Elder Patients of Stroke from the Rural Areas of Shaanxi Province
Source: Int J Environ Res Public Health. 2017 Oct 11;14(10):1211. doi: 10.3390/ijerph14101211 (PMC5664712; doi:10.3390/ijerph14101211)
Supplement: Supplementary file 1 [file ijerph-14-01211-s001.pdf]

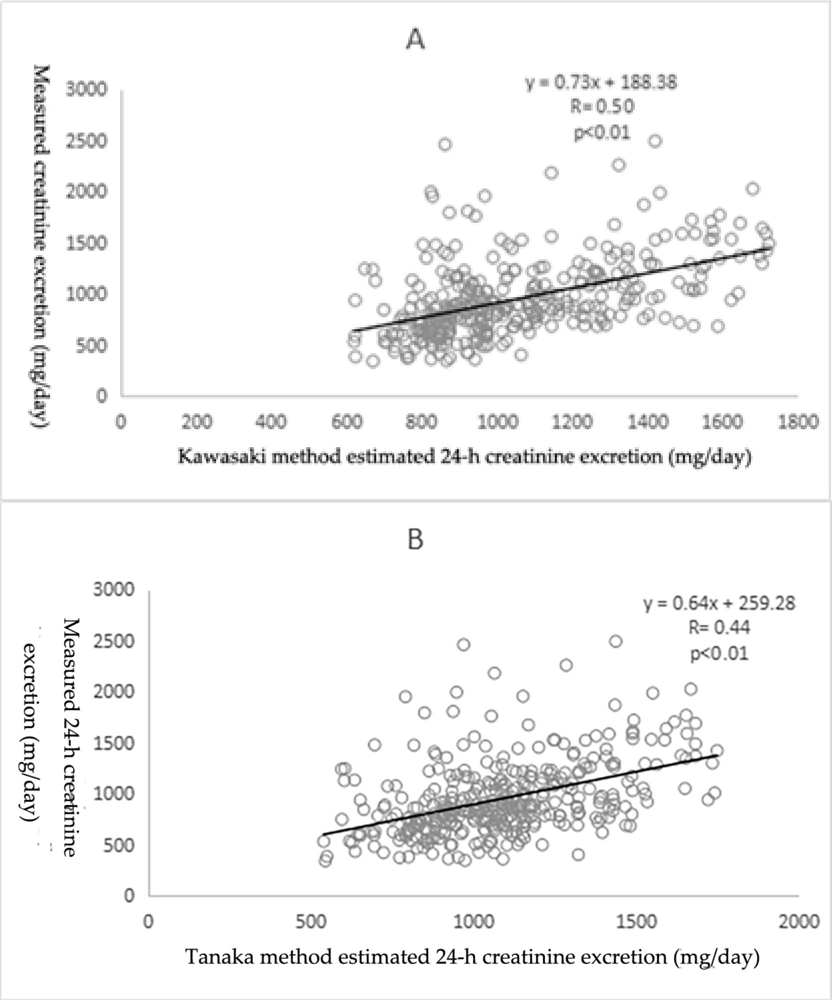

Figure S1. Scatter plots of measured 24-hour creatinine excretion (mg/day) vs Kawasaki. (**A**) and Tanaka; (**B**) methods estimated 24-hour creatinine excretion (mg/day). The hollow circles were scatter points of measured and estimated values. The solid black line was the regression line of the scatters.

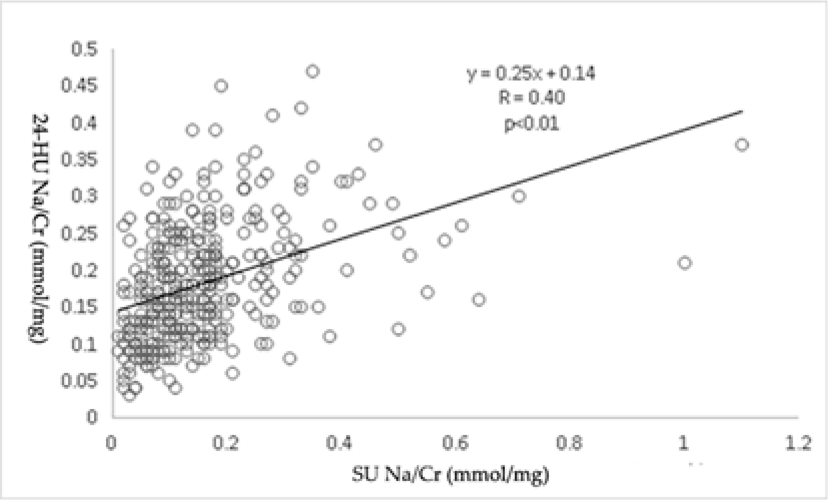

Figure S2. Scatter plots of the ratio of sodium to creatinine (mmol/mg) in spot urine vs the ratio of sodium to creatinine in 24-hour urine (mmol/mg). The hollow circles were scatter points of the ratio values. The solid black line was the regression line of the scatters.
